# Prognostic nutritional index, but not NLR or PLR, is linked to survival in multiple myeloma

**DOI:** 10.3389/fonc.2026.1745422

**Published:** 2026-05-28

**Authors:** Nermin Keni Begendi, Mustafa Duran, Yaşar Culha

**Affiliations:** 1 Division of Hematology, Department of Internal Medicine, School of Medicine, Afyonkarahisar Health Science University, Afyonkarahisar, Türkiye; 2 Division of Medical Oncology, Department of Internal Medicine, Sivas Numune Hospital, Sivas, Türkiye

**Keywords:** inflammatory markers, multiple myeloma, neutrophil-lymphocyte ratio, platelet-lymphocyte ratio, prognosis, prognostic nutritional index

## Abstract

**Background:**

Multiple myeloma (MM) prognosis is influenced by numerous parameters. The prognostic nutritional index (PNI), neutrophil-lymphocyte ratio (NLR), and platelet-lymphocyte ratio (PLR) have emerged as potential prognostic markers in various malignancies.

**Objectives:**

To evaluate the prognostic significance of PNI, NLR, and PLR in predicting overall survival (OS) in newly diagnosed MM patients.

**Methods:**

This retrospective study included 97 patients diagnosed with MM at the Hematology Clinic of Afyonkarahisar Health Sciences University between May 2017 and June 2025. PNI, NLR, and PLR values were calculated from baseline laboratory data at diagnosis. Overall survival was analyzed using Kaplan-Meier curves and Cox proportional hazards regression models.

**Results:**

The median follow-up time was 22.3 months (interquartile range: 6.8–48.0 months). Low PNI (≤34.7) was identified in 38 patients (39.2%). The low PNI group demonstrated significantly higher mortality compared to the high PNI group (76.3% vs. 50%, p = 0.02). Median OS was significantly shorter in the low PNI group compared to the high PNI group (13.5 months vs. 53.3 months, p = 0.007). No significant differences in median OS were observed between low and high PLR or NLR groups. High beta-2 microglobulin (β2M) levels were associated with a 3.19-fold increased risk of death (p = 0.048).

**Conclusion:**

Low PNI at diagnosis is significantly associated with increased mortality and reduced overall survival in MM patients, whereas NLR and PLR do not demonstrate significant prognostic value in this cohort.

## Introduction

Multiple myeloma (MM) is a hematological malignancy characterized by clonal proliferation of plasma cells in the bone marrow. Despite significant advances in therapeutic strategies, including the advent of immunomodulatory drugs, proteasome inhibitors, monoclonal antibodies, and chimeric antigen receptor T-cell (CAR-T) therapy, MM remains largely incurable with high relapse and mortality rates (1). The clinical course of MM is highly heterogeneous, necessitating reliable prognostic markers to stratify patients and guide treatment decisions.

Recent evidence has highlighted the critical interplay between nutrition, inflammation, immunity, and cancer progression. Chronic inflammation and malnutrition are recognized as key contributors to cancer-related morbidity and mortality (2). In MM, several studies have demonstrated a strong association between systemic inflammatory markers and clinical outcomes. Inflammatory indices such as the neutrophil-lymphocyte ratio (NLR) and monocyte-lymphocyte ratio (MLR) have been correlated with survival outcomes in MM patients (2–5). A meta-analysis by Guo et al. revealed that lower baseline NLR and decreasing NLR trends during and after immunotherapy were associated with prolonged survival and improved tumor response in various cancers (6).

In addition to inflammatory markers, nutritional status has emerged as an important prognostic factor in oncology. Several nutritional indices, including the Controlling Nutritional Status (CONUT) score, Nutritional Risk Index (NRI), and Prognostic Nutritional Index (PNI), have been investigated in MM (5, 7–9). The PNI, originally developed by Onodera et al. to assess perioperative nutritional status and surgical risk, is calculated using serum albumin levels and peripheral blood lymphocyte counts [PNI = 10 × serum albumin (g/dL) + 0.005 × total lymphocyte count (per mm³)]. Recent studies have demonstrated that low PNI is an independent risk factor for poor prognosis in newly diagnosed MM patients and is significantly associated with reduced overall survival (5, 7–9).

Similarly, the platelet-lymphocyte ratio (PLR), another inflammatory marker, has been investigated in MM, though results have been inconsistent across studies (10–13). Given the growing body of evidence supporting the prognostic utility of these biomarkers, we aimed to evaluate the prognostic significance of baseline PNI, NLR, and PLR in predicting overall survival in newly diagnosed MM patients treated at our institution.

## Materials and methods

### Study design and patient population

Study design: Retrospective cross-sectional study design

This retrospective cross-sectional cohort study included 97 patients diagnosed with MM at the Hematology Clinic of Afyonkarahisar Health Sciences University between May 2017 and June 2025. The diagnosis of MM was established based on clinical presentation, laboratory findings, bone marrow examination, and imaging studies according to the International Myeloma Working Group (IMWG) criteria.

Inclusion criteria: - Confirmed diagnosis of MM according to IMWG criteria - Age ≥18 years - Availability of complete baseline laboratory data at diagnosis - Initiation of antimyeloma therapy at our institution

Exclusion criteria: - Patients with missing baseline laboratory data - Presence of active infection at diagnosis - Concurrent autoimmune disorders - History of other malignancies within the past 5 years - Patients who did not receive treatment at our institution

### Data collection

Demographic, clinical, and laboratory data were retrospectively collected from electronic medical records. The following parameters were recorded at the time of diagnosis: age, gender, hemoglobin, white blood cell count, absolute neutrophil count, absolute lymphocyte count, platelet count, serum albumin, serum calcium (corrected), lactate dehydrogenase (LDH), creatinine, beta-2 microglobulin (β2M), immunoglobulin type and level, bone marrow plasma cell percentage, and cytogenetic abnormalities (when available).

Disease staging was performed according to the International Staging System (ISS) and Revised International Staging System (R-ISS). Treatment regimens, response to therapy, progression-free survival (PFS), and overall survival (OS) data were also collected.

### Calculation of inflammatory and nutritional indices

The following indices were calculated using baseline laboratory values:

1. Prognostic Nutritional Index (PNI): PNI = 10 × serum albumin (g/dL) + 0.005 × total lymphocyte count (per mm³)
2. Neutrophil-Lymphocyte Ratio (NLR): NLR = absolute neutrophil count / absolute lymphocyte count
3. Platelet-Lymphocyte Ratio (PLR): PLR = platelet count / absolute lymphocyte count

### Statistical analysis

Continuous variables were expressed as median and interquartile range (IQR) or mean ± standard deviation (SD), depending on the distribution. Categorical variables were presented as frequencies and percentages. The normality of continuous variables was assessed using the Kolmogorov-Smirnov test.

Optimal cutoff values for PNI, NLR, and PLR were determined using receiver operating characteristic (ROC) curve analysis with mortality as the endpoint. Patients were then stratified into low and high groups based on these cutoff values.

Survival analyses were performed using the Kaplan-Meier method, and differences between groups were compared using the log-rank test. Univariate and multivariate Cox proportional hazards regression models were used to identify independent prognostic factors for OS. Variables with p < 0.10 in univariate analysis were included in the multivariate model.

All statistical analyses were performed using SPSS version 25.0 (IBM Corp., Armonk, NY, USA). A two-sided p-value < 0.05 was considered statistically significant.

### Ethical considerations

This study was conducted in accordance with the principles of the Declaration of Helsinki. Ethical approval was obtained from the Institutional Review Board of Afyonkarahisar Health Sciences University [Date: 2024-12-13, No: 2024/495]. Due to the retrospective nature of the study, the requirement for informed consent was waived by the ethics committee.

## Results

### Patient characteristics

A total of 97 patients with newly diagnosed MM were included in the analysis. The baseline demographic and clinical characteristics are summarized in **Table 1**. The median age at diagnosis was 67.7 ± 10.3 years (range: [add range]), and 61.9% patients were male. The median follow-up time was 22.3 months (IQR: 6.8–48.0 months). The median follow-up time was 22.3 months (interquartile range: 6.8-48.0 months). Forty patients (41%) had an ECOG 3 performance score. Twenty-seven patients (27.8%) received stem cell transplantation (**Table 2**). While 42.3% of patients received one line of treatment, 8.3% of patients received four or more lines of treatment (**Table 2**). Stable disease response to first-line therapy was observed in 39.2%, while progression was observed in 41.2% (**Table 2**). PNI was 34.7 or less in 38 patients (39.2%), PLR was 149.5 or less in 45 patients (46.4%), and NLR was 4.56 or less in 21 patients (21.6%) (**Tables 2**, **3**).

**Table 1:** Cut-off values determined by ROC analysis for predicting survival.

Continuous variable	AUC (CI)*	Cut-off	*p-*value	Sensitivity (%)	Specificity (%)
PNI	0.667 (0.536-0.737)	34.73	0.02	64.4	64.0
PLR	0.427 (0.291-0.562)	–	0.31	–	–
NLR	0.477 (0.289-0.665)	–	0.81	–	–
Age	0.708 (0.604-0.811)	68.5	<0.01	64.4	71.1
Beta 2 microglobulin	0.657 (0.529-0.784)	5.01	0.02	63.4	58.1
Creatinine	0.658 (0.542-0.774)	0.975	0.01	59.6	61.1
Hemoglobin	0.401 (0.281-0.522)	–	0.12	–	–
Albumin	0.659 (0.544-0.774)	3.83	0.01	75.0	50.0
Calcium	0.393 (0.276-0.410)	–	0.09	–	–

*AUC (CI), area under the curve (confidence interval).

**Table 2:** Demographics and clinical characteristics of the patients.

Characteristics	
Age, mean ± SD*	67.7 ± 10.3
Gender, n(%)	
Female	37 (38.1)
Male	60 (61.9)
ECOG** performance score, n(%)	
PS 0-1	18 (18.6)
PS 2	23 (23.7)
PS 3	40 (41.2)
PS 4	14 (14.4)
CRAB^Ɨ^, n(%)	
1	42 (43.3)
2	36 (37.1)
3 or 4	12 (12.4)
ISS stage, n(%)	
Stage 1	21 (21.6)
Stage 2	31 (32.0)
Stage 3	39 (40.0)
Unknown	6 (6.2)
PBSCT^ǂ^, n(%)	
Absent	68 (70.1)
Present	27 (27.8)
Treatment lines, n(%)	
1	41 (42.3)
2	36 (37.1)
3	10 (10.3)
≥4	8 (8.3)
First-line chemotherapy regimen, n(%)	
VCD^¶^	90 (92.8)
VD^§^	4 (4.1)
Lenalidomide	1 (1.0)
Unknown	2 (2.1)
First-line therapy response, n(%)	
Stabil Disease	38 (39.2)
Remission	16 (16.5)
Progression	40 (41.2)
Unknown	3 (3.1)
PNI, n(%)	
≤34.7	38 (39.2)
>34.7	32 (33.0)
Missing	27 (27.8)
PLR, n(%)	
≤149.5	45 (46.4)
>149.5	25 (25.8)
Missing	27 (27.8)
NLR, n (%)	
≤4.56	21 (21.6)
>4.56	18 (18.6)
Missing	39 (40.2)
Survival status, n (%)	
Exitus	59 (60.8)
Survive	38 (39.2)

*SD, standart deviation; **ECOG, Eastern Cooperative Oncology Group; Ɨ CRAB, creatinine, renal impairment, anemia, bone lesions; ǂ PBSCT, peripheral blood stem cell transplantation; ¶ VCD, bortezomib, cyclophosphamide, dexamethasone; § VD, bortezomib, dexamethasone.

**Table 3:** Patients' clinicopathological characteristics according to PNI.

Variable	PNI ≤ 34.7 (N = 38)	PNI>34.7 (N = 32)	P value
Age, mean ( ± SD*)	69.2 ( ± 10.2)	65.9 ( ± 11.0)	0.20
Gender, n(%)			0.86
Female	15 (39.5)	12 (37.5)	
Male	23 (60.5)	20 (62.5)	
ECOG** performance score, n (%)			0.09
PS 0-1	3 (7.9)	8 (25.0)	
PS 2	10 (26.3)	9 (28.1)	
PS 3	16 (42.3)	13 (40.6)	
PS 4	9 (23.7)	2 (6.3)	
CRAB^Ɨ^ parameters, n(%)			0.58
1	15 (39.5)	15 (48.4)	
2	16 (42.1)	13 (41.9)	
3 or 4	7 (18.4)	3 (9.7)	
ISS stage, n(%)			<0.001
Stage 1	1 (2.6)	16 (24.3)	<0.008^¶^
Stage 2	19 (50.0)	6 (35.7)	0.007
Stage 3	18 (47.4)	10 (40.0)	0.16
PBSCT^ǂ^, n(%)			0.32
Absent	29 (76.3)	21 (65.6)	
Present	9 (23.7)	11 (34.4)	
Treatment lines, n(%)			0.67
1	18 (47.4)	12 (37.5)	
2	14 (36.8)	13 (40.6)	
≥3	6 (15.8)	7 (21.9)	
First line therapy response n (%)			0.68
Stabil disease	19 (51.4)	14 (43.8)	
Remission	4 (10.8)	6 (18.8)	
Progressive disease	14 (37.8)	12 (37.5)	
Hemoglobin, meean (± SD) ALBUMİN	9.54 (± 1.85)	10.96 (± 2.16)	0.04
Albumin, mean (± SD)	2.86 (± 0.42)	4.14 (± 0.66)	<0.001
Creatinine, mean (± SD)	1.76 (± 1.70)	1.56 (± 1.53)	0.62
Calcium, mean (± SD)	8.20 (± 0.92)	9.52 (± 1.01)	<0.001
Survival status			0.02
Exitus	29 (76.3)	16 (50.0)	
Survive	9 (23.7)	16 (50.0)	

*SD, standart deviation; **ECOG, Eastern Cooperative Oncology Group; Ɨ CRAB, creatinine, renal impairment, anemia, bone lesions; ǂ PBSCT, peripheral blood stem cell transplantation; ¶, adjusted new *p*-value is 0.008 for subgroups.

Comparison of groups based on the PNI cut-off value determined by ROC analysis revealed no statistically significant differences in age, gender, ECOG performance score, CRAB parameters, stem cell transplantation, number of treatment lines, treatment responses, or creatinine levels. However, patients in the low PNI group (≤34.7) had significantly lower hemoglobin (p = 0.04), albumin (p < 0.001), and calcium levels (p < 0.001) compared to those with higher PNI. Additionally, the low PNI group exhibited a higher mortality rate (76.3% vs. 50%, p = 0.02).

**Figure 1:**
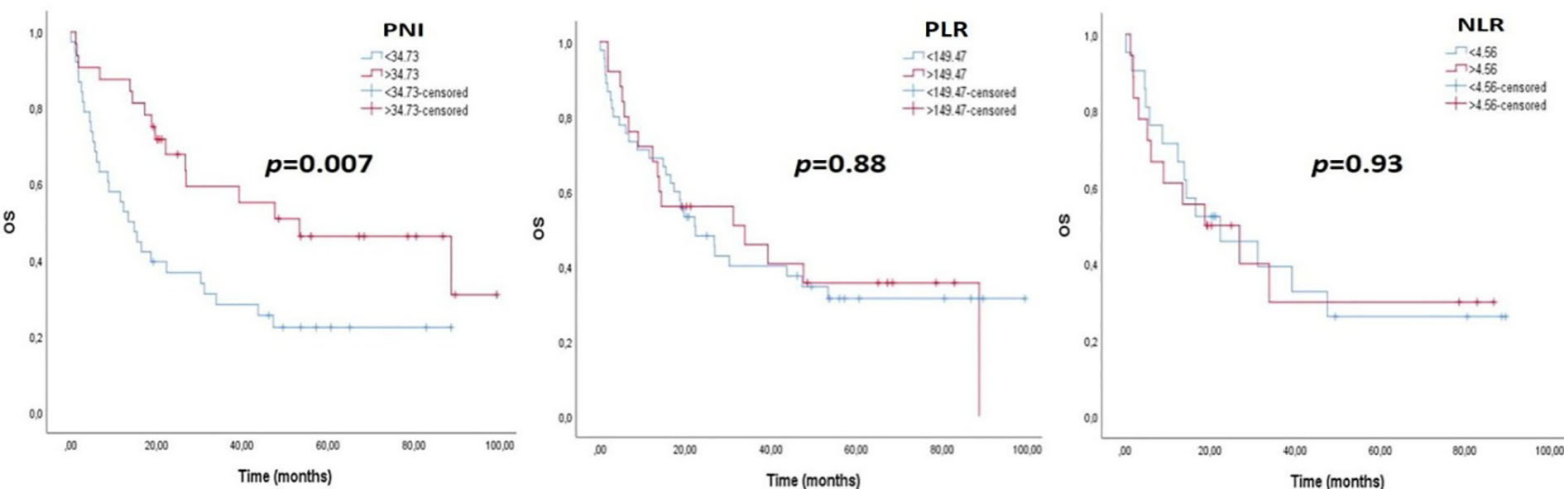
Kaplan-Meier curves according to PNI, TLR, and NLR groups. The median OS was 13.5 months for low PNI and 53.3 months for high PNI. The median OS was 22.3 months for low TLR and 33.9 months for high TLR. The median OS was 22.3 months for low NLR and 18.7 months for high NLR.

In univariate Cox regression analysis, the mortality risk in patients with ISS stage 3 was significantly higher than in patients with stage 1 (HR = 2,95, 95% CI: 1.42-6,14, p<0.01, **Table 4**) There was no statistically significant difference in median overall survival in the higher and lower PLR and NLR group analyses (**Figure 1**). The low PNI group had a 2.84-fold increased risk of death compared to the high group (HR = 2,84, 95% CI: 1.16-6,94, p=0.02, **Table 4**). The higher beta-2 microglobulin level group had a 3.14-fold increased risk of death compared to the lower group (HR = 3.14, 95% CI: 1.01-9.81, p = 0.049; **Table 4**). Median overall survival was 13.5 months in the low PNI group and 53.3 months in the high PNI group (p=0.007, **Figure 1**).

**Table 4:** Univariate and multivariate Cox regression analysis for overall survival.

Variable	Univariate analysis		Multivariate analysis	
	*p-*value	HR* (95,0% CI)	*p*- value	HR* (95,0% CI)
Age (>68 vs ≤68 )	<0.01	2.84 (1.64-4.93)	0.91	1.06 (0.39-2.84)
Hemoglobin (>10.30 vs ≤10.30)	0.02	0.50 (0.27-0.88)	0.67	1.23 (0.49-3.08)
Beta 2 microglobulin (>5.01 vs ≤5.01)	<0.01	3.44 (1.80-6.54)	0.049	3.14 (1.01-9.81)
Albumin (>3.83 vs ≤3.83 )	0.03	0.50 (0.26-0.94)		
Creatinine (>0.975 vs ≤0.975)	0.02	1.96 (1.12-3.44)	0.91	1.05 (0.44-2.53)
Calcium (>8.89 vs ≤8.89 )	0.2	0.70 (0.40-1.22)	-	
ISS stage 2 vs stage 1	0.5	1.31 (0.60-2.86)		
ISS stage 3 vs stage 1	<0.01	2.95 (1.42-6.14)		
Treatment lines (2 lines vs 1 )	<0.01	0.22 (0.12-0.40)	0.01	0.31 (0.12-0.78)
Treatment lines (3 or more lines vs 1)	<0.01	0.13 (0.05-0.32)	0.87	1.14 (0.23-5.63)
PBSCT** (present vs absent)	<0.01	0.14 (0.06-0.33)	0.05	0.21 (0.05-0.99)
Maintenance treatment (present vs absent)	<0.01	0.22 (0.09-0.55)	0.43	0.49 (0.08-2.91)
PLR (>149.5 vs ≤149.5)	0.88	0.95 (0.52-1.76)	-	
NLR (>4.56 vs ≤4.56)	0.93	1.03 (0.47-2.28)	-	
PNI (≤34.73 vs >34.73)	<0.01	2.32 (1.24-4.35)	0.02	2.84 (1.16-6.94)

*HR, hazard ratio; **PBSCT, peripheral blood stem cell transplantation.

## Discussion

This retrospective study evaluated the prognostic significance of PNI, NLR, and PLR in 97 newly diagnosed MM patients. Our findings demonstrate that low PNI at diagnosis is significantly associated with increased mortality and reduced overall survival, whereas NLR and PLR do not show significant prognostic value in this cohort.

The PNI, which integrates both nutritional status (serum albumin) and immune function (lymphocyte count), has emerged as a simple yet powerful prognostic tool in various malignancies (14, 15). Our results are consistent with recent meta-analyses and cohort studies demonstrating that low PNI is an independent predictor of poor outcomes in MM patients (5, 7–9, 15). Zhang et al. reported that MM patients with low PNI had significantly shorter OS and PFS compared to those with high PNI (5). Similarly, Liang et al. found that PNI was independently associated with survival in Chinese MM patients (7).

The prognostic value of PNI in MM may be attributed to several mechanisms. First, hypoalbuminemia reflects not only poor nutritional status but also systemic inflammation and increased tumor burden, both of which are associated with adverse outcomes in MM (2). Second, lymphopenia is a marker of impaired immune function and is associated with disease progression and increased susceptibility to infections, a major cause of morbidity and mortality in MM patients (4). Third, the combination of these two parameters may capture the complex interplay between nutrition, inflammation, and immunity in cancer progression (2, 16).

In contrast to PNI, we did not observe significant associations between NLR or PLR and OS in our cohort. This finding differs from several previous studies that reported NLR and PLR as prognostic markers in MM (10–13). Romano et al. demonstrated that elevated NLR was associated with shorter PFS and OS in newly diagnosed MM patients treated with novel agents (10). Similarly, recent studies have suggested that PLR may predict outcomes in MM patients receiving bortezomib-based regimens (12, 13).

The discrepancy between our findings and previous reports may be attributed to several factors. First, differences in patient populations, including variations in disease stage, treatment regimens, and ethnic backgrounds, may influence the prognostic value of these markers. Second, the optimal cutoff values for NLR and PLR have not been standardized and vary considerably across studies, which may affect their discriminatory power (6, 10–13). Third, our relatively small sample size and single-center design may have limited the statistical power to detect modest associations.

Notably, our study confirmed the well-established prognostic significance of β2M in MM. High β2M levels were associated with a 3.19-fold increased risk of death, consistent with its role as a key component of the ISS and R-ISS staging systems (1). This finding validates the robustness of our dataset and analytical approach.

Our study has several strengths. First, we evaluated multiple inflammatory and nutritional markers simultaneously in the same cohort, allowing for direct comparison of their prognostic utility. Second, we used ROC curve analysis to determine optimal cutoff values specific to our population, enhancing the clinical applicability of our findings. Third, we performed multivariate analysis to adjust for potential confounders and identify independent prognostic factors.

However, several limitations should be acknowledged. First, the retrospective design and single-center nature of the study may introduce selection bias and limit generalizability. Second, the relatively small sample size may have reduced statistical power, particularly for subgroup analyses. Third, we did not have complete cytogenetic data for all patients, which precluded comprehensive R-ISS staging and assessment of high-risk genetic abnormalities. Fourth, longitudinal changes in PNI, NLR, and PLR during treatment were not evaluated, and dynamic changes in these markers may provide additional prognostic information (6). Fifth, we did not assess other nutritional indices such as CONUT or NRI, which have also been reported as prognostic in MM (8, 15).

Future studies should validate our findings in larger, multicenter cohorts with longer follow-up periods and complete cytogenetic data. Prospective studies are needed to evaluate the dynamic changes in PNI and other biomarkers during treatment and their associations with treatment response and survival outcomes. Additionally, the integration of PNI with established prognostic models such as the R-ISS may improve risk stratification and guide personalized treatment strategies in MM patients.

## Conclusion

In this retrospective cohort of 97 newly diagnosed MM patients, low PNI at diagnosis was significantly associated with increased mortality and reduced overall survival, independent of other prognostic factors. In contrast, NLR and PLR did not demonstrate significant prognostic value in our cohort. These findings suggest that PNI, a simple and readily available biomarker reflecting both nutritional status and immune function, may serve as a valuable prognostic tool in MM. Further validation in larger, prospective studies is warranted to confirm these results and explore the potential of PNI-guided interventions to improve outcomes in MM patients.

## Data Availability

The original contributions presented in the study are included in the article/supplementary material. Further inquiries can be directed to the corresponding author.
